# The Relation between Physical Education Teachers’ (De-)Motivating Style, Students’ Motivation, and Students’ Physical Activity: A Multilevel Approach

**DOI:** 10.3390/ijerph18147457

**Published:** 2021-07-13

**Authors:** Nele Van Doren, Katrien De Cocker, Tom De Clerck, Arwen Vangilbergen, Ruben Vanderlinde, Leen Haerens

**Affiliations:** 1 Department of Movement and Sports Sciences, Faculty of Medicine and Health Sciences, Ghent University, 9000 Ghent, Belgium; Katrien.DeCocker@Ugent.be (K.D.C.); Tom.DeClerck@Ugent.be (T.D.C.); Arwen.Vangilbergen@Ugent.be (A.V.); Leen.Haerens@Ugent.be (L.H.); 2 Department of Educational Studies, Faculty of Psychology and Educational Sciences, Ghent University, 9000 Ghent, Belgium; Ruben.Vanderlinde@ugent.be

**Keywords:** Self-Determination Theory, physical education, physical activity, motivating style, controlling style, motivation, gender, lesson topic

## Abstract

Research suggests that physical education (PE) teachers can play a crucial role in the promotion of students’ physical activity. Grounded in Self-Determination Theory, this study investigated how students’ perceptions of PE teachers (de-)motivating style relate to students’ device-based physical activity levels during PE. Moreover, it was examined whether students’ motivation plays an intervening role in this relation and whether students’ physical activity differs according to their gender and lesson topic. A sample of 302 secondary school students aged between 11 and 16 years (M = 13.05, SD = 1.04) completed a questionnaire assessing their perceptions of teachers’ (de-)motivating style and their personal motivation toward PE. Students also wore ActiGraph GT3X accelerometers during the PE lesson. Multilevel structural equation modeling revealed that the teachers’ motivating style had a significant positive relation with students’ autonomous motivation, both at the student level and the class level, and teachers’ controlling style had a significant positive relation with students’ controlled motivation and amotivation at both levels. However, in terms of students’ physical activity levels, students’ gender, the lesson topic, and teachers’ controlling style seemed to be more decisive than students’ motivation and teachers’ motivating style.

## 1. Introduction

The benefits of an active lifestyle during adolescence have been widely demonstrated [1]. For instance, adolescents who are physically active not only perform better at school [2,3], they also sleep better [4] and have less anxiety and depressive symptoms [5]. As active adolescents are more likely to remain physically active in adulthood [6,7,8], they are also expected to gain physical and psychological health benefits in later life. These benefits include reduced risks of cardiovascular diseases, metabolic syndrome, overweight and obesity, cancer, osteoporosis, diabetes type 2, and depression [1,9]. As such, the World Health Organization [10] recommends adolescents to participate in at least 60 min of moderate- to vigorous-intensity physical activity (MVPA) every day. However, many adolescents do not reach this global recommendation [7,11,12]. Moreover, adolescents’ physical activity levels decline rapidly through adolescence, whereby adolescents between the age of 12 and 15 years display the highest decline [13,14]. Therefore, the promotion of physical activity is necessary for this age group. Research suggests that physical education (PE-) teachers can play a crucial role in this promotion [15,16]. One way is to increase physical activity levels during PE. In this respect, the US Health People 2010 recommends students to participate in MVPA for at least 50% of the PE lesson [17]. However, research reveals that most students participate in MVPA for less than 50% of the lesson. For instance, Fairclough and Stratton [18] found that students engage in MVPA between only 27% and 47% of the effective PE lesson time. Furthermore, it appears that students’ physical activity levels during the PE lesson depend on a range of factors such as the goal of the lesson [19], the lesson topic [20], and students’ gender [21]. Indeed, it has been shown that students’ physical activity levels during the PE lesson largely vary according to the lesson topic [20,22]. For example, students displayed lower MVPA levels during racket games when compared to all other topics (i.e., ball games, artistic sports, and fitness training) [20]; lower vigorous physical activity in artistic sports when compared to fitness training [22]; and lower physical activity levels during individual activities when compared to team activities [18,23]. Even though findings are inconsistent across studies, all these studies consistently confirm the variability in percentage time spent in MVPA according to the lesson topic. Furthermore, boys are generally more active than girls [21,24]. The present study aims to build on this work by investigating how secondary school students’ objectively measured physical activity levels differ according to the lesson topic and students’ gender, while also examining relations with students’ motivation [20] and teachers’ (de-)motivating style [25,26].

### 1.1. Students’ Motivation for PE

To examine students’ motivation and teachers’ (de-)motivating style, we rely on Self-Determination Theory (SDT) [27], which is a broad and well-evidenced theory on human behavior and motivation that distinguishes amotivation from controlled motivation and autonomous motivation. Amotivation refers to a complete absence of motivation [27]. An amotivated student feels incapable to engage in PE or claims to have no idea why he/she should participate [20].

Introjected and external regulation are two controlled forms of motivation, as they involve a feeling of pressure, coercion or obligation [20]. Externally regulated students will engage in PE to avoid a punishment or criticism, obtain a reward or appreciation (e.g., gain good grades), or meet external expectations [20,28]. Introjected regulation manifests when students feel pressure that originates from themselves [27]. For example, a student engages in PE to avoid a negative internal state (e.g., guilt, shame) or to gain a positive internal state (e.g., increasing self-esteem, achieving social recognition) [29].

Identified regulation, integrated regulation, and intrinsic motivation constitute autonomous types of motivation because they are volitional in nature [20]. Identified regulation occurs when a student understands the personal relevance of the activity [20,28]. For instance, a student puts effort into the warm-up of the PE lesson when he/she understands that a good warm-up prevents injuries [20]. Integrated regulation is defined as engagement in a behavior as a result of the harmonization of the behavior with the indivudal’s personal values and ideals [20]. For instance, a student engages in PE because he/she values sports, social interaction, and being in good shape [28]. Intrinsic motivation, the highest quality form of motivation, is characterized by interest, enjoyment, satisfaction, and choice [29]. For example, a student engages in PE because he/she finds pleasure and accomplishment in the activity and enjoys the experience of learning new things [30].

These qualitatively different forms of motivation relate differently to important outcomes in PE. Autonomous motivation for PE is associated with higher concentration [31], greater enjoyment [32,33], and more effort [31,32,34]. Controlled motivation and amotivation for PE have been positively related to boredom [32] and unhappiness [33]. In studies using device-based measurements (i.e., heart-rate monitors, pedometers, accelerometers), autonomous motivation for PE is positively related to higher levels of physical activity levels during PE [20] and during leisure time [35], while controlled motivation and amotivation were unrelated to physical activity levels during PE [20] and during leisure time [35]. In comparison to the number of studies investigating the relation between students’ motivation and their physical activity levels using self-reported measures, the number of studies using device-based measurements such as pedometers and accelerometers is scarce [26,36]. Overall, the abovementioned studies stress the importance of enhancing students’ autonomous motivation for PE to achieve higher physical activity levels during PE.

### 1.2. Teachers’ (De-)Motivating Style

According to SDT, a teacher can foster students’ autonomous motivation by adopting a need-supportive motivating style, which is characterized by autonomy-supporting, structuring and relatedness-supportive teaching behaviors [37,38]. When being autonomy-supportive, teachers identify, nurture, and develop students’ interests, preferences, and personal goals [37]. Autonomy-supportive strategies include offering choice [39,40], using inviting language instead of controlling language [41], and accepting students’ input [42]. When providing structure, teachers give students clear information about what to do and how to do it to achieve the desired outcomes [43]. Structuring strategies include communicating transparent expectations [44,45], giving step-by-step guidelines [44,46], and using positive and constructive feedback [47,48]. Finally, when being relatedness-supportive, teachers show noticeable interest and (emotionally) support their students [49]. Relatedness-supportive strategies include being warm and caring, offering affection and unconditional regard, and devoting extensive energy, time, and resources in students [25].

In contrast, a teacher will elicit students’ controlled motivation and amotivation by displaying a need-thwarting motivating style, which is characterized by controlling, chaotic, and cold teaching behaviors [50]. When being controlling, teachers ignore the students’ perspective and instead pressure students to act, think, or feel in a specific way [37]. When being chaotic, teachers state unclear goals, and teachers do no inform students how to achieve these goals. Lastly, when being cold, teachers are unfriendly or even reject or exclude students [51].

In general, SDT-based research suggests that teachers who adopt a motivating style will stimulate their students toward higher engagement [52], health-related well-being [53], and physical activity through the development of autonomous motivation for PE [54,55]. Specifically, an autonomy-supportive style has been related to students’ need satisfaction, autonomous motivation, and positive course-related outcomes in PE, such as effort and exercise intention [56]. In addition, (a small number of) studies in the PE context have related a structuring motivating style to autonomous motivation, enjoyment, perceived importance of PE, and exercise intention [57]. Lastly, studies regarding a relatedness-supportive style are generally scarce. One experimental study found that students’ perceptions of teachers’ relatedness-supportive style were positively related to confidence in their teachers’ ability and enjoyment [58]. However, most studies focused solely on one aspect of a motivating style [59,60], with an autonomy-supportive motivating style being the most commonly investigated. Furthermore, most of these studies fully relied on self-reported measures to assess students’ physical activity levels, risking the issue of shared method variance. To our knowledge, only two studies used accelerometers to determine students’ physical activity levels during PE [25,26]. Both intervention studies, one in elementary school children and the other in secondary school children, showed that positive changes in teachers’ motivating style can increase students’ MVPA during PE.

In addition, studies on teachers’ need-thwarting style have mainly focused on a controlling style, showing that teachers who adopt a controlling style will stimulate students’ controlled motivation and amotivation [52], and maladaptive outcomes, such as fear of failure and less engagement [52,54,61]. Moreover, one study found that students’ perceptions of teachers’ controlling style were negatively related to students’ physical activity levels during leisure time, as measured by accelerometers [62]. Yet, to the best of our knowledge, relations with objectively measured physical activity levels during PE have not been previously examined.

### 1.3. Premise of This Study

The objective of this study is to investigate the relation between teachers’ motivating style (that is an autonomy-supportive, structuring, and relatedness-supporting teaching style), and demotivating style (i.e., controlling) and students’ physical activity levels during PE, and determine if this relation can be indirectly explained by the quality of students’ motivation toward PE (i.e., autonomous, controlled, amotivation). In doing so, this study builds on previous research by (1) focusing on all aspects of a teachers’ motivating style, (2) including an aspect of teachers’ need-thwarting style, (3) assessing physical activity levels during the PE lesson by means of devices (accelerometers), and (3) taking both the lesson topic as well as students’ gender into account.

Based on SDT [27] and previous research findings [20,46,53,63], multiple hypotheses are put forward: we expect that when students perceive their teacher as more need-supportive, their autonomous motivation for PE will be fostered, while their controlled motivation and amotivation for PE will be lower (H1a). When a teacher is perceived to be more controlling, we hypothesize that students’ controlled motivation and amotivation will be fostered, while their autonomous motivation will be lower (H1b). In turn, we expect students’ autonomous motivation will relate to higher physical activity levels during the PE lesson (H2). Theoretically, we would expect that students’ controlled motivation and amotivation will relate to lower physical activity levels during the PE lesson. Yet, empirical evidence does not show such relations. Therefore, we examine relations between controlled motivation and amotivation and physical activity in a more explorative manner. In line with previous research, we further expect boys to be more physically active than girls (H3), and we will explore how students’ physical activity varies according to the lesson topic. In addressing these hypotheses, we decomposed the variance at the student level (i.e., individual) and the class level (i.e., contextual), because the extent to which students experience the teacher as need-supportive is likely to depend on both individual (e.g., their personality) and teacher level (e.g., how need-supportive the teacher actually is) factors. Such decomposement allows examining at the student level how individual students’ perceptions of the teacher style relate to their personal motivation for PE and in turn their activity levels. At the same time, it allows to examine at the class level whether classes are on average more active, more autonomously motivated, and less controlled motivated and amotivated when teachers are generally perceived as more need-supportive and less controlling.

## 2. Materials and Methods

### 2.1. Participants and Data Collection

For this cross-sectional study, 260 Flemish secondary schools were contacted by e-mail or telephone. When the PE teachers were interested in participating in this study, the teacher was provided with extra information by e-mail, telephone, or personal contact. If the teacher agreed to participate, one of their classes was chosen to participate in the study. In total, 29 PE teachers from 22 different secondary schools participated in this study (8.46% response rate), of which 18 teachers were men (62.07%) and 11 teachers were female (37.93%). Teachers who provided their age and years of experience (N = 16) were on average 39.5 (SD = 11.38) years old and had on average 17.66 (SD = 11.52) years experience. For every PE teacher, one class from grades 8 to 10 was chosen, with students’ age ranging between 11 and 16 years. In total, 302 students participated in this study. Of the 299 students who provided their sex (N = 299), 159 were boys (53.18%) and 140 were girls (46.82%). Students providing their age (N = 228) were on average 13.05 years old (SD = 1.04). The average number of students per class was 10.41 (SD = 3.86). The lesson topics could be grouped in one of four categories [20]: ball games (e.g., volleyball, handball, soccer; 7 classes, or 24.14%), artistic sports (e.g., gymnastics, dance, rope skipping; 6 classes, or 20.69%), fitness training (e.g., running, fitness track; 11 classes, or 37.93%), and racket games (badminton, table tennis; 5 classes, or 17.24%). All participating teachers and students, as well as the parents of the students, signed an informed consent form. The study was approved by the Ethical Committee of Ghent University (EC: 2017/0213).

All participating PE teachers filled out a short questionnaire that provided information regarding their sex, age, and years of experience. At the start of the PE lesson, the students were mounted with an accelerometer. Since the number of available accelerometers was limited (N = 15), accelerometers were randomly assigned to students who signed the informed consent form and were present on the day of the study. Students wore the accelerometer on their right hip using an elastic belt. At the end of the PE lesson, the students who wore an accelerometer were asked to individually fill out a set of questionnaires to determine their gender, age, motivation for PE, and their perceptions of the teachers’ (de-)motivating style. It took about 10 to 15 min for students to fill out the questionnaire.

### 2.2. Measures

Students’ situational motivation for PE was assessed using the validated Behavioral Regulations in Physical Education Questionnaire (BRPEQ) [20]. The BRPEQ is an adapted Dutch version of the Behavioral Regulations in Exercise Questionnaire (BREQ-II) [64] and consists of 20 items that were rated on a five-point Likert scale ranging from 1 (completely disagree) to 5 (completely agree). Eight items were used to determine autonomous motivation. For example: “I put effort in this PE lesson because I liked this PE lesson”. While SDT proposes autonomous motivation consists of three different regulations (i.e., identified regulation, integrated regulation, and intrinsic motivation), we did not collect data on integrated regulation, as the BRPEQ only measures identified regulation and intrinsic motivation. Another eight items measured controlled motivation, such as “I put effort in this PE lesson because I would feel guilty if I didn’t put effort in the PE lesson”. Lastly, four items referred to amotivation. For instance: “I don’t see why I would put effort in this PE lesson”. The Cronbach’s alpha for autonomous motivation, controlled motivation, and amotivation was respectively 0.85, 0.76, and 0.66.

To determine students’ perceptions of teachers’ (de-)motivating style, students were asked to fill out the Dutch version of Teacher as Social Context Questionnaire (TASCQ) [65,66] and the Psychologically Controlling Teaching (PCT) [50], respectively. The translation followed the guidelines of the International Test Commission [67] and has previously been used and validated in other studies [54,61]. The TASCQ measures autonomy-support (6 items), structure (5 items), and relatedness-support (6 items), while the PCT measures control (9 items). All items had to be answered on a five-point Likert scale ranging from 1 (completely disagree) to 5 (completely agree). An example of an autonomy-support item is “During this PE lesson, my teacher gave me lots of choices about how I can deal with the exercises”. An example of a structure item is “During this PE lesson, my teacher clarified what he/she expects of me”. An example of a relatedness-support item is “My teacher likes me”. An example of a controlling item is “During the PE lesson, my teacher made me feel guilty when I dissatisfied him/her”. The Cronbach’s alpha was 0.71 for autonomy-support, 0.69 for structure, 0.78 for relatedness-support, and 0.77 for control.

To determine students’ physical activity during PE, Actigraph GT3x (+) accelerometers were used. Actigraph accelerometers are known to be valid and reliable measures to objectively assess duration, frequency, and intensity of physical activity among youth [68,69]. Furthermore, Actigraph GT3x accelerometers are omnidirectional accelerometers that are sensitive to movements in all three axes. The Actigraph GT3x accelerometer detects movements over pre-specified time periods called epochs that were set on fifteen-second intervals. Movements within each epoch are converted to ‘activity counts’ with the use of the ‘Actilife’ programme. Then, these activity counts are interpreted to determine minutes spent at different activity intensities (e.g., moderate and vigorous) by using cut-off points. The current study focused on MVPA because this type of physical activity is recommended for public health [15,17]. Similar to previous research [21,62], the cut-off points for physical activity of Evenson et al. [70] were used to determine MVPA (>2296). To determine MVPA during the PE lesson, the raw scores were converted into percentage of time spent in MVPA by dividing the raw scores by lesson time. In Belgium, PE is grouped in one or two blocks of 50 min. For all participating classes, class schedules were consulted. Time spent in MVPA was calculated as a percentage of the 50 or 100 min’ lesson hours, which included the time spent to go to the gym and to get changed as well as the actual lesson time. By using percentages, comparison between classes was made possible, since the duration of the PE lessons was different from one lesson to another (either 50 min or 100 min, respectively 18 and 11 classes).

### 2.3. Data Analyses

To further check the validity of the measures (BRPEQ, TASCQ, and PCT), confirmatory factor analysis (CFA) was used in Mplus [71]. Amotivation consisted of 4 items, autonomous and controlled motivation each consisted of 8 items, teachers’ motivating style consisted of 17 items, and teachers’ controlling style consisted of 9 items. Model fit was determined by using normed chi-square (normed χ2), root mean square error of approximation (RMSEA), comparative fit index (CFI), and standardized root-mean-square residual (SRMR). For a good model fit, normed χ2 should be < 2; RMSEA < 0.05 (< 0.08 is acceptable), CFI > 0.95 (> 0.90 is acceptable), and SRMR < 0.05 (< 0.08 is acceptable) [72]. CFA revealed an acceptable to good model fit for three out of four parameters (χ2 = 1717.83, df = 979; CFI = 0.80; RMSEA = 0.05; SRMR = 0.07).

Preliminary analyses were executed using SPSS 25.0 (descriptive statistics and bivariate Pearson correlations); and similar to previous studies [54], structural equation modeling (SEM) in Mplus [71] was used to investigate the research questions. Given the nested structure of the data, multilevel SEM analyses were conducted, with students at the first level and classes (or teachers) at the second level. To determine the model fit of the SEM model, the same fit indices as with the CFA were used [72]. First, a null model was estimated to evaluate how much of the variation in percentage of lesson time spent in MVPA could be attributed to both levels (student and teacher). Thereafter, student gender and lesson topic were included separately into the null model to evaluate the relation between both predictors and MVPA. To account for the lesson topic, three dummy variables were created (i.e., artistic sports, fitness training, and racket games) and contrasted against the reference group (ball games). To find out which lesson topics were significantly differed from one other, all possible group comparisons were performed by changing the reference group. Second, a multilevel SEM model was tested. It was decided to use a two-level model, since the school and class level were largely confounded (e.g., for 20 out of the 22 schools, the number of teachers per school was N = 1). Furthermore, a three-level model did not yield a better fit than a two-level model, and the variance at the school level was zero. Therefore, using a three-level model did not seem justified, and the data were treated as a two-level model. In this full model, the indirect effect of the teachers’ (de-)motivating style onto the percentage of lesson time spent in MVPA via students’ autonomous motivation, controlled motivation, and amotivation was tested. In this full model, both individual students’ perceptions of the (de-)motivating teaching style and motivations, as well as average class perceptions of the (de-)motivating teaching style and motivations were included as respectively group-mean centered and grand-mean centered variables. This allowed examining relations at the student level as well as at the class level. The full model was controlled for students’ gender and for lesson topic. For all these analyses, a *p*-value inferior to 0.05 was considered statistically significant, and a *p*-value inferior to 0.10 was considered to reveal a trend toward significance.

Due to missing data for age in 74 of 302 students, we did not include students’ age as a covariate in the full model. In order to estimate the stability of the full model without students’ age, we decided to conduct sensitivity analyses by comparing this full model with a model controlled for students’ age, hereby relying on multiple imputations for handling the missing data for age. Multiple imputations are considered one of the most highly recommended methods for dealing with missing data [73]. In line with previous research, five imputed datasets were created, stored, and analyzed [74]. Subsequently, results are combined according to the rules suggested by Rubin [75].

## 3. Results

### 3.1. Preliminary Analysis

Descriptive statistics (means, standard deviations) and bivariate Pearson correlations are shown in Table 1. In Supplementary Tables S1–S6, correlation coefficients between the study variables are presented separately for boys and girls, and per lesson topic. On average, the students engaged in MVPA during 19.03% (SD = 10.87%; 9.52 min per lesson) of the lesson, with only 3.31% of the students achieving the recommended amounts of MVPA during the PE lesson (2.65% of the boys and 0.66% of the girls). In addition, correlations showed that autonomy-support, structure, and relatedness-support were significantly and positively associated with autonomous motivation, while these were negatively correlated with amotivation. Only autonomy-support was significantly and positively related to controlled motivation, and structure showed a trend toward a significant positive correlation with controlled motivation. Furthermore, a controlling style was negatively associated with autonomous motivation, and it was positively correlated with controlled motivation and amotivation. Moreover, small but significant positive relations were found between students’ autonomous motivation and students’ MVPA during PE on the one hand and between teachers’ autonomy-support and students’ MVPA during PE on the other hand. Lastly, a trend toward a significant relation was found between teachers’ relatedness-suport and students’ MVPA during PE (see Table 1).

### 3.2. Main Analysis

A two-level null model for percentage of lesson time spent in MVPA was estimated and revealed that 21.61% of the variability was accounted for by student differences and 78.39% was accounted for by class differences. Inclusion of gender revealed that boys (22.63%, SE = 2.21) displayed a significantly higher percentage of time spent in MVPA than girls (18.78%, SE = 1.21). Student gender explained 9% of the student differences. Second, the relation between the lesson topics and students’ MVPA levels was investigated. The lesson topic explained 17.32% of the class differences. Specifically, the mean percentage of lesson time spent in MVPA in the multilevel model was 28.12% (SE = 5.12) during ball games, 24.05% (SE = 3.61) during artistic sports, 24.60% (SE = 1.77) during fitness training, and 25.48% (SE = 1.52) during racket games. Students were significantly less active during fitness training when compared to ball games (*p* < 0.05; see Table 2), although the differences between ball games and racket games displayed a trend toward significance, with students being more active in ball games (*p* = 0.08).

Next, direct relations between teachers’ (de-)motivating style and students’ physical activity levels at both the student and class level were tested (hereby controlling for gender and lesson topic). For this model, a good fit was obtained (χ2 = 0.00, df = 0; CFI = 1.00; RMSEA < 0.001; SRMR within < 0.001; SRMR between < 0.001). The relations between teachers’ motivating style and students’ physical activity levels was significant on neither level (student level: β = −0.71; *p* = 0.18; class level: β = 0.83; *p* = 0.89). The relation between teachers’ controlling style and students’ physical activity levels was not significant on the student level (β = 0.62; *p* = 0.37). However, this relation was significant and negative on the class level (β = −13.31; *p* = 0.02)

**Figure 1:**
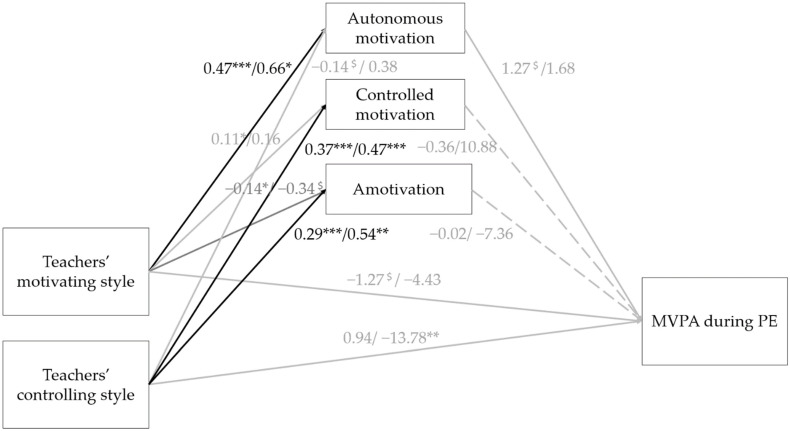
Full model with teachers’ (de-)motivating style as a possible predictor for students’ MVPA during PE when taking both students’ gender and lesson topic into account. βs are presented at both the student level (first number) and the class level (β student level/β class level); ^$^ *p* < 0.1; * *p* < 0.05; ** *p* < 0.01; *** *p* < 0.001; MVPA moderate- to vigorous-intensity physical activity; PE physical education. Black lines indicate a significant relation on both the student and class level, while gray full lines indicate only a significant or trend to significant relation on one of the two levels. Finally, gray dashed lines indicate that no significant relations were found.

Finally, students’ motivation (at both levels) was included in the multilevel model (see Figure 1). Results indicated that teachers’ motivating style was significantly and positively related to autonomous motivation at both the student (β = 0.47; *p* < 0.001) and class level (β = 0.66; *p* = 0.02). Teachers’ motivating style was also significantly and positively related to controlled motivation at the student level (β = 0.11; *p* = 0.03), but not at the class level (β = 0.16; *p* = 0.51). Teachers’ motivating style was significantly and negatively related to amotivation at the student level (β = −0.14; *p* = 0.05), and it displayed a trend toward a significant negative relation with amotivation at the class level (β = −0.34; *p* = 0.08). Furthermore, teachers’ controlling style was significantly and positively related to controlled motivation and amotivation at both the student (controlled motivation: β = 0.37; *p* < 0.001; amotivation: β = 0.29; *p* < 0.001) and class level (controlled motivation: β = 0.47; *p* < 0.001; amotivation: β = 0.54; *p* = 0.001). Teachers’ controlling style displayed a trend toward a significant negative relation with autonomous motivation at the student level (β = −0.14; *p* = 0.06), yet it was not significantly related to autonomous motivation at the class level (β = −0.38; *p* = 0.26).

Autonomous motivation showed a trend toward a significant positive relation with students’ percentage of the lesson time spent in MVPA on the student level (β = 1.27; *p* = 0.10) but not at the class level (β = 1.05; *p* = 0.84). However, none of the indirect effects via autonomous motivation were significant. Moreover, controlled motivation and amotivation were not associated with the percentage of lesson time spent in MVPA neither at the student level nor at the class level. Thus, also the indirect effects via controlled motivation and amotivation were not significant. Surprisingly, in the full model, teachers’ motivating style displayed a trend toward a significant negative relation with students’ percentage of the lesson time spent in MVPA on the student level (β = −1.27; *p* = 0.06). Only the direct relation between teachers’ controlling style and students’ percentage of lesson time spent in MVPA remained significant on the class level (β = −13.78; *p* = 0.001). Yet, these results need to be interpreted with care, since no good model fit was obtained (χ2 = 66.19, df = 6; CFI = 0.75; RMSEA = 0.18; SRMR within = 0.07; SRMR between = 0.06).

### 3.3. Sensitivity Analyses

A two-level model that controlled for students’ age was estimated and revealed similar results when compared to the abovementioned results, confirming the stability of the presented model. Only two small differences were found on the student level: teachers’ motivating style now displayed a significant negative relation with students’ physical activity levels during PE (β = −1.28; *p* = 0.05), and the *p*-value displaying the relation between students’ autonomous motivation and their physical activity levels during PE was now 0.09 instead of 0.10 (β = 1.26). In Supplementary Figure S1, the model controlled for students’ age is shown.

## 4. Discussion

Given the low physical activity levels in adolescence, the promotion of physical activity is a big concern worldwide in this age group [10]. Research suggests that PE teachers can play a crucial role in this promotion [15,16]. Grounded in SDT, this study investigated how students’ perceptions of PE teachers (de-)motivating style relate to their motivation for PE and in turn to their physical activity levels during the PE lesson as measured by means of device-based measures. Moreover, it was also examined how students’ gender and the lesson topic relate to students’ physical activity levels during PE. Overall, the results suggest that teachers’ motivating style is positively related to students’ autonomous motivation for PE both at student and class level. Teachers’ motivating style is also negatively related to students’ amotivation for PE at the student level and showed a trend toward a significant negative relation to students’ amotivation at the class level. Furthermore, teachers’ controlling style is positively related to students’ controlled motivation and amotivation for PE on both levels. These results are in line with the theoretical tenets of SDT [27]. In terms of students’ physical activity levels, students’ gender, the lesson topic, and teachers’ controlling style seemed to be more decisive than students’ motivation and teachers’ motivating style.

### 4.1. Physical Activity Levels during the PE Lesson

Results revealed that students engaged in MVPA for only 19.03% of the PE lesson on average, which is even lower than the percentages found in previous reviews (e.g., 34.7%, [76]; 46.8%, [18]). Furthermore, only 3.31% of present students scored equal to or above 50%, which is also lower in comparison to previous studies (e.g., 12.8%, [20]). These results show that this study sample does not meet the recommended amount of MVPA during PE (50% of the PE lesson, [17]). It is important to note that percentage time spent in MVPA was calculated consulting the class schedule, hereby including time spent to go to the gym and to get changed. Therefore, percentages found in this study are lower compared to other studies [18,76]. In the study of Lonsdale et al. [26], percentage time spent in MVPA was calculated by recording start and finish times of each lesson as indicated by the school bell. This method is similar to the method used in this study, and similar results were found.

In addition, the estimated model including students’ physical activity levels and the topic of the lesson revealed that there were significant differences between students’ physical activity levels during the lesson, implying that albeit being taught by the same teacher, students’ physical activity levels differed from one another. Yet, our analyses also showed that class differences (78.39%) outweighed student differences (21.61%). This suggests that not only students’ personal characteristics have to be taken into account (e.g., students’ gender), but particularly class or teacher-related variables will explain differences in activity levels (e.g., lesson topic). Aelterman et al. [20] also reported similar distributions with 63% of the variability in MVPA being accounted for by the class level in their study.

### 4.2. Relations between Teachers’ Motivating Style, Students’ Motivation, and Students’ Physical Activity Levels during PE

In the current study, we investigated the relation between teachers’ (de-)motivating style and students’ motivation. In line with our hypothesis, teachers’ motivating style was significantly and positively related to students’ autonomous motivation both at the student and the class level. This is an important finding as SDT-related research in PE highlights the importance of enhancing students’ autonomous motivation for PE to achieve positive outcomes, such as higher concentration [31], greater enjoyment [32,33], and more effort [31,32,34]. Moreover, teachers’ motivating style displayed a significant negative relation with students’ amotivation on the student level, and it showed a trend toward a negative relation on the class level. In addition, teachers’ controlling style was significantly and positively related to students’ controlled motivation and amotivation on both levels. This reveals that it is important to minimize teachers’ controlling style and optimize teachers’ motivating style, since controlled motivation and amotivation for PE is related to negative outcomes, such as boredom [32] and unhappiness [33]. Moreover, these results stress the importance of examining both teachers’ motivating and demotivating styles. This is shown in the relatively unique and differential pathways between teachers’ motivating and controlling style and students’ motivation, with teachers’ motivating style fostering a bright pathway and teachers’ controlling style supporting a dark pathway [54].

Furthermore, as relations were found both at the student and at the class level, we can conclude that it is not only when students personally perceive their teachers as more motivating or controlling that their personal motivation is affected. There also appear to be class-level relations or contextual effect. When teachers are generally perceived as more motivating, their classes display higher levels of autonomous motivation. When teachers are generally perceived as more controlling, their classes display higher levels of controlled motivation and amotivation for PE. These findings suggests that teachers’ (de-)motivating style is not only important for students’ personal motivation but is equally important for the classes’ motivation in general.

In addition to these relations between teachers’ (de-)motivating style and students’ motivation, an important question was whether these variables were related to students’ physical activity levels during PE. In the presented model, only a trend toward a significant positive relation was found betweens students’ autonomous motivation and their MVPA during PE on the student level. Although we hypothesized, based on previous research [54,55], that teachers who adopt a motivating style will stimulate their students toward being physically active through the development of autonomous motivation for PE, this assumption was only partially confirmed by our model. Note that the correlation table displayed a significant positive relation between students’ autonomous motivation, teachers’ autonomy support, and students’ physical activity levels, and a trend toward a significant relation between teachers’ relatedness support and students’ physical activity levels. This suggests that students display higher levels of physical activity when they are autonomously motivated and when they perceive their teachers as more autonomy and relatedness supportive. Yet, these relations do not hold in the full model (when accounting for students’ gender, lesson topic, and the hierarchical structure of the data); therefore, no indirect relation was obtained. In the full model, the direct relation between a need-supportive teaching style and MVPA during PE even became negative on the student level. In line with the findings of the full model, a recently conducted intervention study in elementary school children also showed that enhanced perceived support from teachers did not affect students’ physical activity levels [77]. These findings contrast prior research that found positive relations between teachers’ motivating style [25] or students’ autonomous motivation and physical activity levels during PE [20]. Moreover, relations between students’ controlled motivation and amotivation and students’ physical activity levels during PE were not significant. Although, we expected, based on theoretical insights that amotivated students and controlled motivated students would be less active, this assumption was not confirmed by our model. However, this finding is in line with prior empirical research using device-based measures [20].

A significant direct relation was found between teachers’ controlling style and students’ physical activity levels during PE at the class level, with classes perceiving their teachers as more controlling displaying lower percentages of lesson time spent in MVPA. One could assume that students would have no choice but being active with a controlling teacher. Yet, this appeared not to be the case. Previous research on teachers’ controlling style mainly focused on relations with physical activity during leisure time and not physical activity during PE. In line with the findings of the current research, Koka et al. [62] found a significant negative relation between teachers’ controlling style and students’ MVPA levels during leisure time (as measured by means of accelerometers). On the other hand, Rodrigues et al. [78] found no significant direct relation between teachers thwarting style (i.e., controlling, chaotic, and cold) and students’ intention to continue exercising.

### 4.3. Students’ Physical Activity Levels According to Students’ Gender and Lesson Topic

Results of the current study confirmed that students’ physical activity levels vary according to students’ gender. In line with our expectations, we found that boys engaged in more MVPA than girls. This finding is in line with many studies that also revealed that girls were less active during the PE lessons [21,24]. Furthermore, students’ gender explained 9% of the variability in students’ physical activity levels during PE, which is in line with the study of Aelterman et al. (6%, [20]).

Moreover, lesson topic accounted for 17.32% of the class differences, which is in line with the findings of Aelterman et al. [20], who reported that lesson topic accounted for 19% of the class differences. In addition, students in the present study accumulated more MVPA during ball games. While only differences between ball games and fitness training reached significance, an inspection of the averages suggests that ball games differed from the three other lesson topics. The unequal distribution of the topics with an under-representation of racket games and artistic sports may have influenced our results. Overall, this finding favoring ball games in terms of students’ activity levels is in line with the study by Erwin et al. [23]. This study found that students displayed higher physical activity levels during team activities in comparison to individual activities, and Fairclough and Stratton [18] showed that students’ physical activity levels were the highest during team games in comparison to individual games, individual activities, and movement activities. However, in contrast to our findings, Aelterman et al. [20] found that students particularly engaged in less MVPA during racket games when compared to all other topics (i.e., ball games, artistic sports, and fitness training). Delextrat et al. [22] showed that students engaged in more MVPA during fitness training compared to artistic sports, while no such differences were found in the current study. Even though findings clearly differ across studies, all these studies consistently confirm the variability in percentage time spent in MVPA according to the lesson topic.

It appeared in the current study that lesson topic is more decisive for students’ MVPA levels during PE than teachers’ motivating style. In contrast to our expectations, we even found a trend toward a significant negative relation between teachers’ motivating style and students’ activity levels at the student level. To our knowledge, only one other study investigated teachers’ motivating style in relation to students’ physical activity levels during PE while taking the lesson topic into account [23]. These authors showed that lesson topic may interact with teachers’ style as students displayed the highest physical activity levels in lessons with team activities without the provision of choice as well as in lessons with individual activities with the provision of choice. The correlations of the current study also revealed that particularly teachers’ autonomy-support is related to students’ physical activity levels. Thus, it is possible that certain motivating strategies such as offering choice are of larger influence for students’ physical activity levels than others. Secondly, it appears that certain motivating strategies exert a different effect depending on lesson topic [23], which is an issue that is worth further exploration.

### 4.4. Practical Implications

A major concern rising from the results of this study is that students spent very low percentages of time in MVPA during the PE lesson. If increasing students’ MVPA levels during the lesson is the sole goal, it might be worthwhile to consider ball games, as students appear to accumulate more MVPA during these activities. Yet, it is important to acknowledge that PE has many other goals that are equally important. For instance, a major goal of PE is to enhance students’ personal and social skills [79], which may not necessarily be achieved more easily during ball games when compared to other lesson topics. Teachers are also recommended to adopt a motivating style to benefit both students’ and classes’ autonomous motivation for PE, while at the same time minimizing their controlling style to minimize students’ and classes’ controlled motivation and amotivation. Thus, future intervention studies should not only focus on developing teachers’ motivating style (an autonomy-supportive, structuring, and relatedness-supportive style), but should also raise awareness among teachers about the detrimental effects of a teachers’ controlling style and discourage teachers from adopting such a style.

### 4.5. Limitations and Future Directions

First, the study used a cross-sectional study design. As such, the observed relations between teachers’ (de-)motivating style and students’ motivation during PE might be interpreted in both ways. For example, students who are more autonomously motivated are more positive toward the teachers’ approach, while students with higher controlled motivation and amotivation are more negative toward the teachers’ approach. Future research could use a longitudinal study design to investigate the causal and long-term effects of a teachers’ motivating style on student’s physical activity levels.

Secondly, it is worth mentioning that selection bias might have occurred, since the accelerometers were randomly given to students who were present in the class and had their informed consent form with them. Therefore, students that were eager to participate in this research may have been more likely to be selected in this research. By no means can the sample of the current study be considered representative of the entire population.

Thirdly, only 228 out of 302 students provided information regarding their age. Therefore, we were not able to include students’ age in the full model in our study. Sensitivity analyses indicated that the full model was similar to a model in which the missing data regarding students’ age were imputed, hereby relying on multiple imputations. While this method is recommended in case of missing data, this method has a few pitfalls, with multiple imputations assuming that missing data are missing at random being the main pitfall [80].

Finally, in the multilevel SEM analyses, we focused on teachers’ motivating style as a whole. Future research should opt to investigate the relation between specific motivating styles and/or strategies and students’ physical activity levels during PE. Moreover, future research could also add other teachers’ demotivating styles (e.g., chaos and coldness) to the model.

## 5. Conclusions

In conclusion, results suggest that teachers’ motivating style is positively related to students’ autonomous motivation at both the student and the class level, and teachers’ controlling style is positively related to students’ controlled motivation and amotivation on both levels. However, in terms of students’ physical activity levels during PE, students’ gender, the lesson topic, and teachers’ controlling style seemed to be more decisive than students’ motivation and teachers’ motivating style.

## Figures and Tables

**Table 1:** Descriptive statistics and bivariate Pearson correlations.

Variable	N	Mean (SD)	2	3	4	5	6	7	8	9	10
1. Percentage of MVPA during PE	302	19.03 (10.87)	0.17 **	0.04	−0.09	0.17 **	−0.02	0.10 ^$^	0.09	−0.08	−0.02
2. Autonomous motivation	302	3.83 (0.73)		0.09	−0.41 **	0.39 **	0.32 **	0.46 **	0.45 **	−0.17 **	−0.23 **
3. Controlled motivation	302	1.77 (0.61)			0.29 **	0.15 *	0.06	0.04	0.10 ^$^	0.38 **	−0.08
4. Amotivation	302	1.53 (0.65)				−0.14 *	−0.16 **	−0.20 **	−0.19 **	0.30 **	0.08
5. Perceived teachers’ autonomy-support	301	3.30 (0.77)					0.55 **	0.63 **	0.85 **	−0.02	−0.04
6. Perceived teachers’ structure	301	3.51 (0.80)						0.60 **	0.85 **	−0.01	−0.02
7. Perceived teachers’ relatedness-support	301	3.58 (0.70)							0.86 **	−0.15 *	−0.05
8. Perceived teachers’ motivating style	301	3.47 (0.65)								−0.07	−0.04
9. Perceived teachers’ controlling style	301	1.71 (0.60)									−0.04
10. Students’ age	228	13.05 (1.04)									

** Correlation is significant at the 0.01 level; * Correlation is significant at the 0.05 level; ^$^ correlation showed a trend towards significance at the 0.10 level; MVPA moderate- to vigorous-intensity physical activity, PE physical education.

**Table 2:** Means and standard deviations in percentages of MVPA (percentage of class time) during PE as a function of lesson topic.

Lesson Topic	M (SE)	β
Ball games	28.12 (5.12) ^a^	
Artistic sports	24.05 (3.61) ^a,b^	−4.07
Fitness training	24.60 (1.77) ^b^	−3.51 *
Racket games	25.78 (1.51) ^a,b^	−2.64

MVPA moderate- to vigorous-intensity physical activity, PE physical education. Note. Values in parentheses are standard errors. * *p* < 0.05. Regression equations were repeated several times by changing the reference category to obtain coefficients for all combinations of lesson topic. A mean is significantly different from another mean (*p* < 0.05) if they have different superscripts.

## Data Availability

Data gathered in this study are not available for public access, since the informed consents did not ask for permission to share data publicly. Data remain confidential according to the ethical approval process of the Committee for Medical Ethics and is held on secure and password protected servers.

## Supplementary Materials

The following are available online at https://www.mdpi.com/article/10.3390/ijerph18147457/s1, Figure S1: Full model with teachers’ (de-)motivating style as a possible predictor for students’ MVPA during PE, when taking students’ gender, students’ age and lesson topic into account (using imutations), Table S1: Descriptive statistics and bivariate Pearson correlations for boys; Table S2: Descriptive statistics and bivariate Pearson correlations for girls; Table S3: Descriptive statistics and bivariate Pearson correlations for ball games; Table S4: Descriptive statistics and bivariate Pearson correlations for artistic sports; Table S5: Descriptive statistics and bivariate Pearson correlations for fitness training; Table S6: Descriptive statistics and bivariate Pearson correlations for racket games.
