# Supplementary material for: The Relation between Physical Education Teachers’ (De-)Motivating Style, Students’ Motivation, and Students’ Physical Activity: A Multilevel Approach
Source: Int J Environ Res Public Health. 2021 Jul 13;18(14):7457. doi: 10.3390/ijerph18147457 (PMC8307004; doi:10.3390/ijerph18147457)
Supplement: Supplementary file 1 [file ijerph-18-07457-s001.zip › ijerph-1261886-supplementary.pdf]

Table S1. Descriptive statistics and bivariate Pearson correlations for boys.

Variable	N	Mean (SD)	2	3	4	5	6	7	8	9	10
1. Percentage of MVPA during PE	159	19.99 (12.50)	.19*	.09	-.07	.23**	.01	.13	.14$	-.05	-.06
2. Autonomous motivation	159	3.84 (.73)		.04	-.47**	.33**	.28**	.39**	.39**	-.27**	-.26**
3. Controlled motivation	159	1.80 (.64)			.34**	.22**	.08	.10	.16*	.38**	-.08
4. Amotivation	159	1.60 (.70)				-.08	-.16$	-.17*	-.15$	.33**	.10
5. Perceived teachers' autonomy-support	158	3.27 (.81)					.56**	.65**	.86**	.01	-.07
6. Perceived teachers' structure	158	3.45 (.81)						.64**	.85**	.02	.06
7. Perceived teachers' relatedness-support	158	3.54 (.73)							.88**	-.13	-.08
8. Perceived teachers' motivating style	158	3.42 (.68)								-.04	-.04
9. Perceived teachers' controlling style	158	1.83 (.65)									-.02
10. Students' age	123	13.09 (1.06)									

**Correlation is significant at the 0.01 level; *Correlation is significant at the 0.05 level; $ correlation showed a trend towards significance at the 0.10 level; MVPA moderate- to vigorous-intensity physical activity, PE physical education.

Table S2. Descriptive statistics and bivariate Pearson correlations for girls.

Variable	N	Mean (SD)	2	3	4	5	6	7	8	9	10
1. Percentage of MVPA during PE	140	18.10 (8.63)	.11	-.08	-.14$	.04	-.09	.05	-.01	-.22**	.06
2. Autonomous motivation	140	3.82 (.73)		.15$	-.34**	.45**	.35**	.53**	.52**	-.04	-.20*
3. Controlled motivation	140	1.72 (.58)			.19**	.05*	.03	-.01	.03	.36**	-.10
4. Amotivation	140	1.43 (.57)				-.21*	-.14**	-.21*	-.21**	.17**	.04
5. Perceived teachers' autonomy-support	140	3.35 (.71)					.54**	.59**	.84**	-.06	.05
6. Perceived teachers' structure	140	3.58 (.78)						.55**	.84**	-.01	-.10
7. Perceived teachers' relatedness-support	140	3.64 (.66)							.84**	-.13*	-.01
8. Perceived teachers' motivating style	140	3.52 (.60)								-.07	-.03
9. Perceived teachers' controlling style	140	1.57 (.50)									-.12
10. Students' age	105	13.01 (1.01)									

**Correlation is significant at the 0.01 level; *Correlation is significant at the 0.05 level; $ correlation showed a trend towards significance at the 0.10 level; MVPA moderate- to vigorous-intensity physical activity, PE physical education.

Table S3. Descriptive statistics and bivariate Pearson correlations for ball games.

Variable	N	Mean (SD)	2	3	4	5	6	7	8	9	10
1. Percentage of MVPA during PE	74	26.23 (13.87)	.08	.03	.03	.05	-.11	-.03	.-.03	0.04	.39**
2. Autonomous motivation	74	3.96 (.62)		-.13	-.35**	.31**	.24*	.34**	.34**	-.33**	-.25$
3. Controlled motivation	74	1.91 (.73)			.41**	.04	.04	-.04	.01	.44**	-.13
4. Amotivation	74	1.45 (.67)				-.05	.02	-.07	-.04	.36**	-.11
5. Perceived teachers' autonomy-support	74	3.67 (.80)					.56**	.65**	.86**	-.07	-.08
6. Perceived teachers' structure	74	3.75 (.77)						.63**	.85**	-.1	-.12
7. Perceived teachers' relatedness-support	74	3.83 (.67)							.87**	-.12	-.16
8. Perceived teachers' motivating style	74	3.75 (.64)								-.11	-.14
9. Perceived teachers' controlling style	74	1.64 (.58)									-.03
10. Students' age	48	12.79 (1.05)									

**Correlation is significant at the 0.01 level; *Correlation is significant at the 0.05 level; $ correlation showed a trend towards significance at the 0.10 level; MVPA moderate- to vigorous-intensity physical activity, PE physical education.

Table S4 Descriptive statistics and bivariate Pearson correlations for artistic sports.

Variable	N	Mean (SD)	2	3	4	5	6	7	8	9	10
1. Percentage of MVPA during PE	69	16.65 (11.04)	.35**	-.03	-.29*	.13	-.06	.14	.08	-.14	.24$
2. Autonomous motivation	69	3.90 (.76)		.07	-.50**	.32**	.20	.39**	.34**	-.15	-.24$
3. Controlled motivation	69	1.55 (.48)			.10	.12	.06	.13	.12	.21$	.02
4. Amotivation	69	1.51 (.61)				-.23$	-.15	-.29*	-.25*	.23$	.27*
5. Perceived teachers' autonomy-support	68	3.26 (.72)					.61**	.67**	.86**	-.08	.11
6. Perceived teachers' structure	68	3.66 (.73)						.74**	.89**	.14	.16
7. Perceived teachers' relatedness-support	68	3.66(.71)							.91**	-.04	.06
8. Perceived teachers' motivating style	68	3.52 (.64)								.01	.13
9. Perceived teachers' controlling style	68	1.59 (.49)									-.11
10. Students' age	59	13.39 (.93)									

**Correlation is significant at the 0.01 level; *Correlation is significant at the 0.05 level; $ correlation showed a trend towards significance at the 0.10 level; MVPA moderate- to vigorous-intensity physical activity, PE physical education.

Table S5. Descriptive statistics and bivariate Pearson correlations for fitness training.

Variable	N	Mean (SD)	2	3	4	5	6	7	8	9	10
1. Percentage of MVPA during PE	119	16.89 (6.50)	-.14	-.06	.05	.01	-.09	-.04	-.05	-.11	-.12
2. Autonomous motivation	119	3.68 (.76)		.27**	-.35**	.44**	.39**	.52**	.54**	-.04	-.16
3. Controlled motivation	119	1.77 (.58)			.28**	.19*	.08	.08	.14	.44**	.06
4. Amotivation	119	1.61 (.67)				-.11	-.20*	-.15$	-.19*	.33**	.08
5. Perceived teachers' autonomy-support	119	3.12 (.71)					.53**	.59**	.84**	.01	-.01
6. Perceived teachers' structure	119	3.28 (.86)						.49**	.84**	.03	-.14
7. Perceived teachers' relatedness-support	119	3.42 (.70)							.82**	-.20*	-.04
8. Perceived teachers' motivating style	119	3.27 (.63)								-.06	-.08
9. Perceived teachers' controlling style	119	1.71 (.59)									-.03
10. Students' age	88	12.93 (.98)									

**Correlation is significant at the 0.01 level; *Correlation is significant at the 0.05 level; $ correlation showed a trend towards significance at the 0.10 level; MVPA moderate- to vigorous-intensity physical activity, PE physical education.

Table S6. Descriptive statistics and bivariate Pearson correlations for racket games.

Variable	N	Mean (SD)	2	3	4	5	6	7	8	9	10
1. Percentage of MVPA during PE	40	16.17 (9.02)	.56**	-.08	-.24	.19	-.26	.11	.04	-.16	-.69**
2. Autonomous motivation	40	3.89 (.71)		.01	-.51**	.37*	.17	.44**	.41**	-.37*	-.55**
3. Controlled motivation	40	1.84 (.56)			.44**	.16	.03	-.05	.07	.26	-.17
4. Amotivation	40	1.43 (.56)				-.13	-.25	-.42**	-.33*	.34*	.13
5. Perceived teachers' autonomy-support	40	3.23 (.75)					.36*	.47**	.81**	.16	-.17
6. Perceived teachers' structure	40	3.48 (.56)						.54**	.76**	.02	.18
7. Perceived teachers' relatedness-support	40	3.47 (.60)							.82**	-.04	-.12
8. Perceived teachers' motivating style	40	3.39 (.51)								.07	-.05
9. Perceived teachers' controlling style	40	2.04 (.69)									.01
10. Students' age	33	13.15 (1.20)									

**Correlation is significant at the 0.01 level; *Correlation is significant at the 0.05 level; MVPA moderate- to vigorous-intensity physical activity, PE physical education.

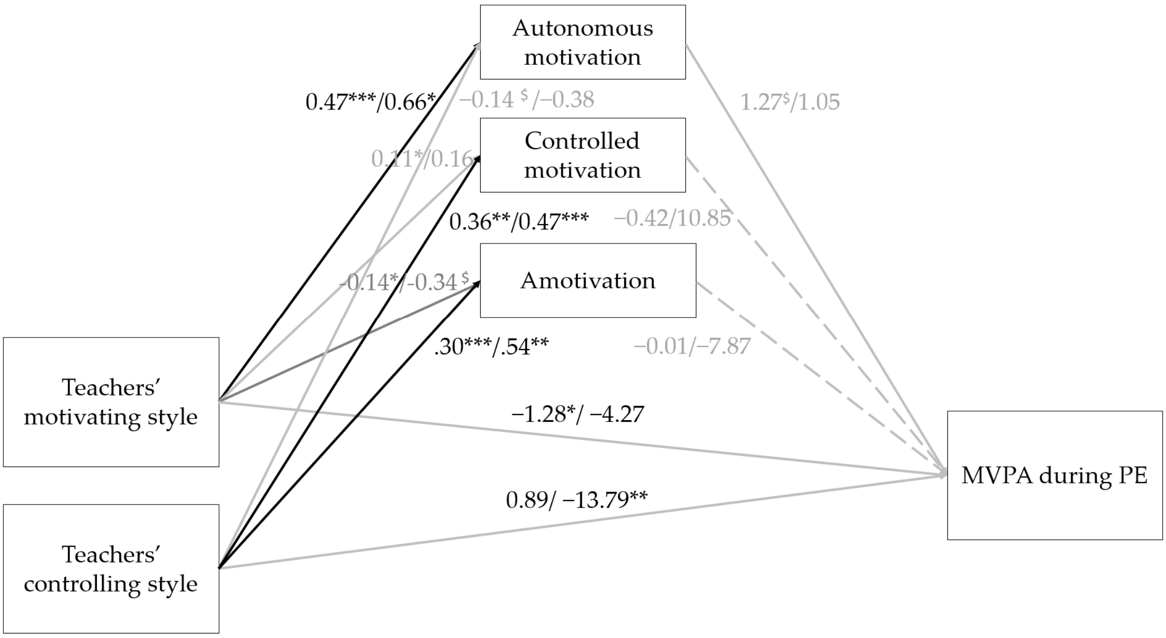

Figure S1. Full model with teachers' (de-)motivating style as a possible predictor for students' MVPA during PE, when taking students' gender, students' age and lesson topic into account (using imutations).

βs are presented at both the student-level (first number) and the class-level (βstudent-level/βclass-level); $^{\$}p < 0.1$; $^{*}p < 0.05$; $^{**}p < 0.01$; $^{***}p < 0.001$; MVPA moderate- to vigorous-intensity physical activity; PE physical education.
